# Noise Induces Hopping between NF-κB Entrainment Modes

**DOI:** 10.1016/j.cels.2016.11.014

**Published:** 2016-12-21

**Authors:** Mathias Heltberg, Ryan A. Kellogg, Sandeep Krishna, Savaş Tay, Mogens H. Jensen

**Affiliations:** 1 Niels Bohr Institute, University of Copenhagen, 2100 Copenhagen, Denmark; 2 Department of Biosystems Science and Engineering, ETH Zürich, 8092 Zürich, Switzerland; 3 Simons Center for the Study of Living Machines, National Center for Biological Sciences, Bangalore 560065, Karnataka, India; 4 Institute for Molecular Engineering, University of Chicago, Chicago, IL 60637, USA; 5 Institute for Genomics and Systems Biology, University of Chicago, Chicago, IL 60637, USA

## Abstract

Oscillations and noise drive many processes in biology, but how both affect the activity of the transcription factor nuclear factor κB (NF-κB) is not understood. Here, we observe that when NF-κB oscillations are entrained by periodic tumor necrosis factor (TNF) inputs in experiments, NF-κB exhibits jumps between frequency modes, a phenomenon we call “cellular mode-hopping.” By comparing stochastic simulations of NF-κB oscillations to deterministic simulations conducted inside and outside the chaotic regime of parameter space, we show that noise facilitates mode-hopping in all regimes. However, when the deterministic system is driven by chaotic dynamics, hops between modes are erratic and short-lived, whereas in experiments, the system spends several periods in one entrainment mode before hopping and rarely visits more than two modes. The experimental behavior matches our simulations of noise-induced mode-hopping outside the chaotic regime. We suggest that mode-hopping is a mechanism by which different NF-κB-dependent genes under frequency control can be expressed at different times.

## Graphical abstract

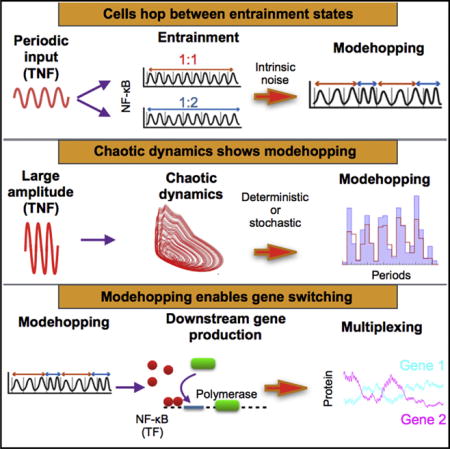

## INTRODUCTION

Oscillation is a conserved dynamic feature of many biological systems. Increasingly oscillation is appreciated to play a role in transcriptional processes in the living cell, given the large number of transcriptional regulators now observed to exhibit oscillation or pulsing (Levine et al., 2013; Gonze et al., 2002). Noise is a core feature of biological systems, and it impacts variability and timing of oscillatory transcriptional regulators (Eldar and Elowitz, 2010; Elowitz et al., 2002). However, the roles of oscillation and noise in gene regulation are still incompletely understood.

**Figure 1:**
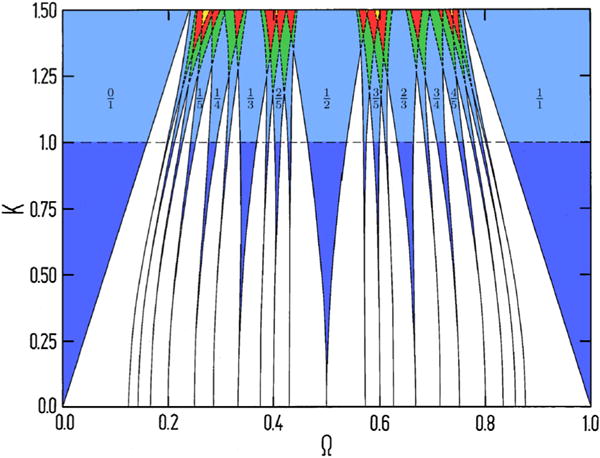
Schematic Diagram of Arnold Tongues On the horizontal axis is the frequency (Ω) of the external (TNF) oscillator and the vertical axis is its amplitude (K). The blue regions are ones in which the internal and external oscillators are entrained, the numbers attached to each region describes the frequency ratio for the entrainment. The white regions show intermixed quasi-periodic and periodic behavior, too finely intermingled to be separated by our plot. The dashed line indicate where the tiniest tongues start to overlap. The green, red, and yellow regions show overlapping behavior, but now also including a chaotic element (Jensen et al., 1984).

Periodic inputs may lead to entrainment of oscillators, a phenomenon where the oscillatory process locks, in frequency and phase, to the external signal. Canonical examples of entrainment in physics include pendulum clocks and lasers; in these systems there exists well-developed theory describing how two oscillators can couple in the way that one external (that is, an independent periodic input) couples to an internal oscillator. The output of the internal oscillator depends on the coupling to the external and to the difference in frequency between the two. When they couple, we call it entrainment, and these regions of entrainment grow with increasing amplitude of the external oscillator. This is depicted schematically in Figure 1. On the horizontal axis is the frequency of the external oscillator (here tumor necrosis factor [TNF]) while on the vertical axis is it is amplitude. These entrainment regions are called Arnold tongues (Jensen et al., 1984); they are indicated as regions of green, red, and yellow. In the case of entrainment between the internal (here nuclear factor κB [NF-κB]) and external oscillator, we observe the widening of the tongues.

**Figure 2:**
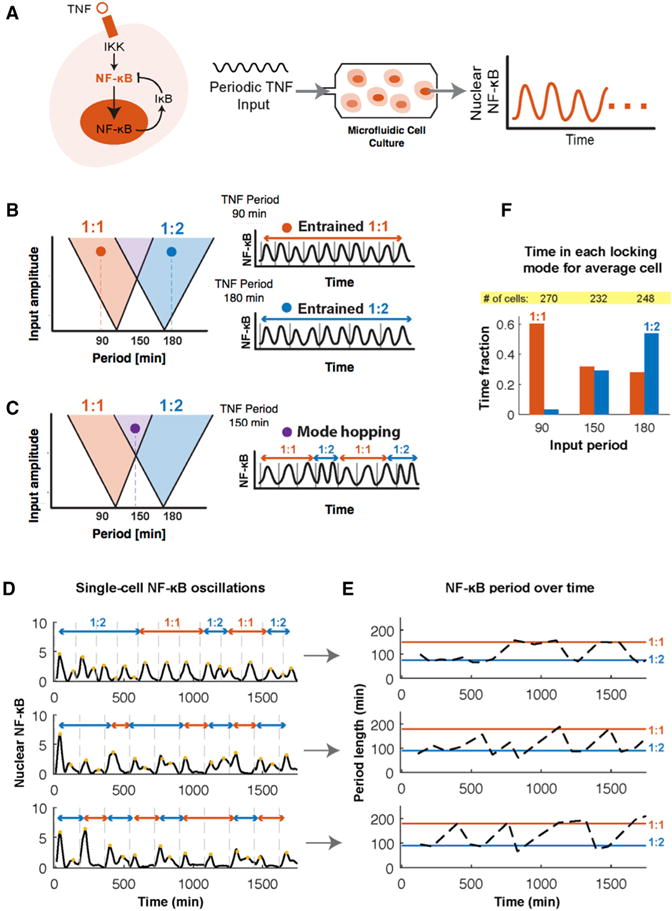
Experimentally Measured Frequency Jumps (Mode-Hopping) in NF-κB Oscillations (A) TNF activates IKK and NF-κB, causing IκB negative feedback leading to oscillations in NF-κB nuclear translocation. We apply periodic TNF input using microfluidics and monitor nuclear NF-κB oscillation dynamics using live cell fluorescence imaging. (B) Periodic forcing of an oscillator leads to entrainment visualized by regions called Arnold tongues. In the 1:1 NF-κB entrainment mode (orange region), the NF-κB period matches the 90-min period of the fluctuating TNF input. In the 1:2 entrainment mode (blue region), there is one TNF input cycle for every two cycles of the NF-κB oscillation. (C) In overlapping Arnold tongue regions, multiple entrainment modes are possible. Here, noise may enable spontaneous transitions between entrainment modes as observed in experiments. (D) Three examples of mode-hopping in single-cell NF-κB traces. Timing of TNF input is indicated by vertical gray dashed lines. (E) Plots of NF-κB oscillation period versus time. Colors indicate intervals in entrainment 1:1 (orange) and 1:2 (blue) modes. (F) Comparison of time an average cell spends in each entrainment mode, for differing TNF input frequencies.

However, it is unclear whether biological oscillators can exhibit behaviors that are similarly complex. Recently, it was shown in single mammalian cells that periodic cytokine inputs entrain the nuclear localization oscillations of NF-κB (Kellogg and Tay, 2015) (schematized in Figure 2A), a transcription factor that plays a central role in environmental sensing and the immune response. In this earlier work, noise (i.e., the dynamic variability in molecular interactions), was demonstrated to synergistically enhance the ability of NF-κB oscillations to entrain to periodic cytokine input from the environment. Specifically, it was observed that noise increased NF-κB oscillation amplitude and gene expression under periodic stimulation. Nonetheless, how noise interacts with both the periodic input and the oscillator itself to mediate entrainment in signaling networks like NF-κB is not yet clear. Here, we demonstrate that noise facilitates a phenomenon we call “mode-hopping”: NF-κB oscillations remain entrained but switch spontaneously between two frequencies. This phenomenon qualitatively resembles mode-hopping behavior observed in lasers, another form of oscillator (Mork et al., 1990). We suggest that mode-hopping may diversify the expression patterns of frequency-modulated genes.

## RESULTS

### Mode-Hopping in Entrained NF-κB Oscillations under Fluctuating TNF Input

In cells, the extracellular ligand TNF initiates a series of intracellular processes that can produce oscillations in the concentration of nuclear NF-κB under specific conditions. Specifically, TNF activates IκB kinase (IKK), which causes the NF-κB transcription factor to enter the cell nucleus and regulate gene expression including upregulation of IκB negative feedback (schematized in Figure 2A) (Krishna et al., 2006). When TNF is delivered periodically, the fraction of NF-κB localized within the nucleus oscillates with a natural oscillation period of 90–100 min (Kellogg and Tay, 2015). Accordingly, we previously showed that NF-κB oscillations can synchronize across the population and entrain cells to the TNF input, and noise was found to facilitate entrainment and efficient gene expression (Kellogg and Tay, 2015). For example, TNF with a 180-min period can entrain NF-κB at a 90-min period for a 1:2 (input:oscillator) locking or entrainment mode (schematized in Figure 2B) (original observation described in Kellogg and Tay [2015]). These regions are defined as Arnold tongues, and the entrainment is schematized in Figure 2B.

Our current investigation began with repeating the observations described in Kellogg and Tay (2015). Using microfluidic cell culture, we delivered periodic TNF stimulation to fibroblasts and recorded dynamics of NF-κB nuclear localization by live cell fluorescence microscopy. Not only did we confirm that NF-κB oscillations occur, we also observed that they show spontaneous frequency jumps and transitions between locking modes during the time course of periodic stimulation for some external forcing periods (Figure 2C). Specifically, these transitions involve apparent spontaneous changes in amplitude (Figure 2D) and doubling and halving of the oscillation frequency over time (Figure 2E); borrowing from the literature on Martin et al. (1997), we refer to this phenomenon as mode-hopping. Compared to 90 min TNF input where cells entrain almost exclusively at the 1:1 locking mode, at 150 min, the average cell spends equal time in 1:1 and 1:2 modes. For 180 min input, the average cell spends the most time in the 2:1 mode but also ~30% of time in the 1:1 mode (Figure 2F). The appearance of multiple entrainment modes during the time course suggests that the system is in the overlapping Arnold tongue regions (schematized in Figures 2B and 2D). It is understood in physics that in noise-free (i.e., deterministic) systems, spontaneous transitions between locking modes cannot occur before the multiple overlaps within the Arnold tongue regions cause a transition into chaos (Jensen et al., 1984). However, whether and how transitions between entrainment modes can spontaneously occur in a noisy system like the NF-κB network is not clear; if these transitions occur, it is not clear what drives them. To answer these questions, we turned to simulation.

### Noise Induces NF-κB Frequency Jumps between Entrainment Modes

**Figure 3:**
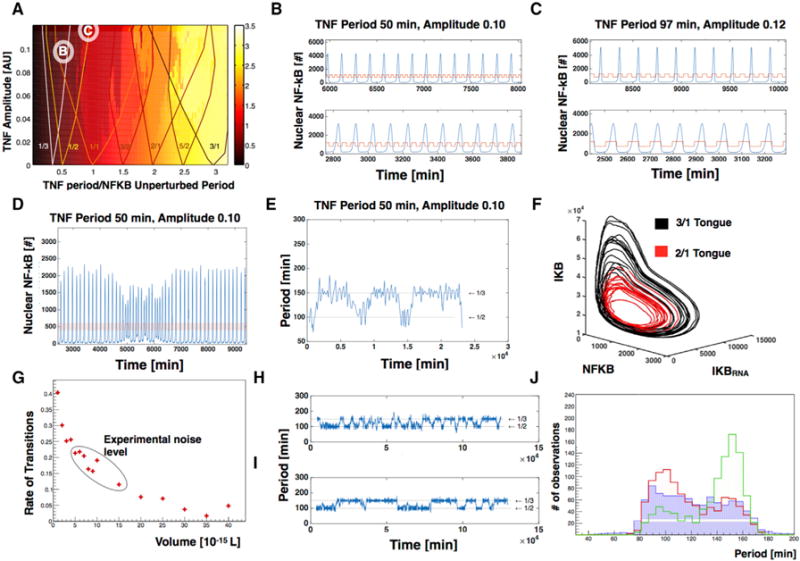
Noise Induces Mode-Hopping in Overlapping Arnold Tongue Regions (A) Arnold tongue diagram for a deterministic model of NF-κB oscillations driven by a periodic square pulse of TNF. Note that the amplitude is dimensionless. The colors show the ratio of the observed NF-κB frequency to the driving TNF frequency as defined in the color bar (right) (Jensen and Krishna 2012). (B and C) Deterministic simulations of NF-κB behavior conducted within regions of parameter space that exist within the overlapping region between Arnold tongues. The simulations shown in (B) were conducted using the parameters in the region of space labeled “B” in Figure 2A, the simulations shown in (C) were conducted using the parameters in the region of space labeled “C” in Figure 2A. Red traces indicate TNF input frequency (50 min in B, 97 min in C; all amplitudes are 0.1 AU); blue traces describe the behavior of NF-κB. (D) Stochastic (Gillespie) simulation of NF-κB behavior conducted within region of parameter space labeled “B” in Figure 2A. Red traces indicate TNF input frequency (period of 50 min; amplitude of 0.1 AU); blue traces describe the be the behavior of NF-κB. (E) Additional visualization of the data shown in (D) where the period between successive NF-κB peaks is plotted as a function of time. The horizontal lines correspond to integer multiples of the time period of the driving TNF oscillation. (F) The trajectories of individual simulations conducted as in (D), plotted in a phase space that describes IKB, IKB_RNA_, and NK-kB values. Colors indicate the different entrained states the trajectory visits. (G) The number of transitions between frequency modes per thousand oscillations as a function of simulation volume; simulated noise decreases with increasing volume; data are taken from simulations analogous to the one shown in (D) but conducted at different cell volumes. The rate of transitions that corresponds to what is found in the experiments are shown in the red circle. (H and I) Additional visualizations of the data shown in (G) where the period between successive NF-κB peaks is plotted as a function of time. The horizontal lines correspond to integer multiples of the time period of the driving TNF oscillation. (J) The distribution of periods is shown, and we see that they peak around integer multiples of the TNF period, and when noise decreases, the system spends longer times in the high period sta.

First, we considered the differential equations model (Jensen and Krishna, 2012), described in the STAR Methods, that captures the essential features of NF-κB behavior. We started by deterministically simulating this model’s behavior in response to oscillatory inputs of different periods and amplitudes; in aggregate, these simulations define the Arnold tongue regions of this model within parameter space. For the NF-κB system, the structure of the tongues is not symmetric, and overlapping regimes of tongues start at quite low amplitudes (Jensen and Krishna, 2012; Kellogg and Tay, 2015) (Figure 3A). As expected, we observed that when NF-κB oscillations are simulated deterministically within the overlapping Arnold tongue regions of parameter space (as defined by the amplitude and frequency of external oscillator), oscillations settle in different entrained states depending on the initial conditions (Figures 3B and 3C; see the STAR Methods for details of the simulations). Mathematically speaking, this means that more than one limit cycle exists, and depending on the basins of attraction, a trajectory decay be attracted to one of the limit cycles. Mode-hopping was, however, not observed, because transitions between stable states do not occur for the deterministic system. This is at the very heart of deterministic systems; once an initial position is defined, that trajectory will be followed, and if the system is inside a basin of attraction, it cannot leave this state.

Next, we considered whether noise could mediate mode-hopping and frequency jumps in NF-κB oscillations by adding noise to our simulations using the Gillespie algorithm (Gillespie, 1977) while keeping the average concentrations of NF-κB and other molecules the same. In these stochastic simulations, we find that transitions between entrained states do indeed occur and show mode-hopping events similar to experimental observations (Figures 3D). These transitions are more easily observed if we look at the periods, where it is clear that they make transitions between states (Figure 3E). Another way to study this phenomena is to look at the trajectory in the three-dimensional phase space spanned by NF-κB, I-κB mRNA, and I-κB (the variables in the first three differential equations presented in the STAR Methods). This presents a quite intuitive way of thinking of the mode-hopping; the trajectory oscillates with two different radii, and this gives rise to the two different periods of oscillation (Figure 3F). Based on these analyses, we conclude that noise mediates hopping between entrainment modes and could serve as a mechanism in the cell to produce quick switching of NF-κB oscillation frequency.

To understand this observation in a more detailed way, we simulated different noise levels by controlling the volume and number of molecules in the simulation. We find that as noise is increased (smaller simulation volume and hence smaller number of molecules), mode-hopping transitions happen more often with more entrainment modes visited. In this sense, increasing the noise tends to broaden the Arnold tongues of the system (Figures 3G–3I). Systems with little noise, in contrast, usually spend very long times in one entrained state, and we find that the system tends to be more in a high period state for small noise compared to large noise. We also find that systems with high noise jump quickly and spend approximately the same time in each entrained state (Figure 3J). Together, these simulations demonstrate that noise is able to reproduce the mode-hopping frequency transitions that we observe in experiments. The mode-hopping seen in the overlapping tongue region is reminiscent of the noise-induced hopping one would observe in a classical bistable system but with the states defined by frequencies and amplitudes of oscillations. Next, we investigated whether mode-hopping is restricted to stochastic systems in the early overlapping regime, or systems operating close to the chaotic regime may also exhibit mode-hopping within the Arnold tongue regions and how this was related to the (deterministic)transition into chaos.

### Mode-Hopping Is a Characteristic Feature for Noisy and Chaotic Systems

**Figure 4:**
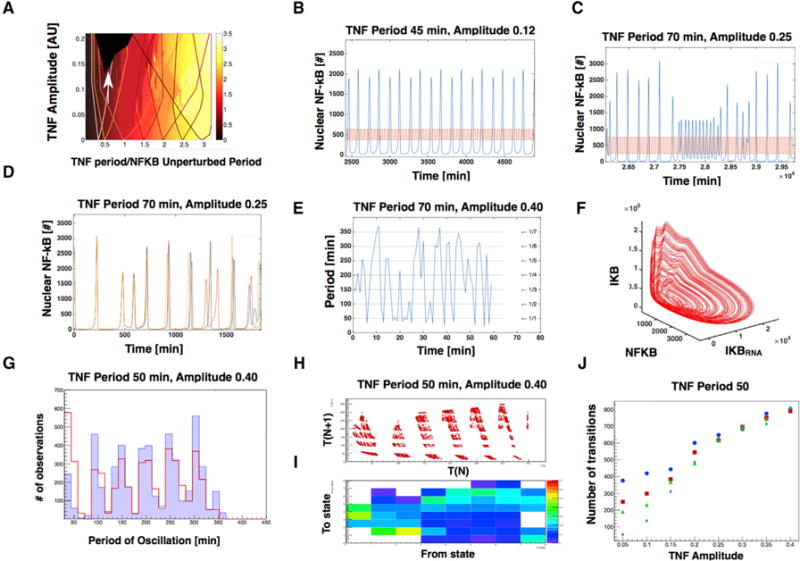
Deterministic Chaos in NF-κB Oscillation Manifests as Mode-Hopping (A) Arnold tongue diagram for a deterministic model of NF-κB, same as Figure 2A, but with TNF amplitude spanning a larger range, including the onset of chaos (black section, indicated by the white arrow). (B and C) Before the onset of chaos, interesting phenomena arise for the deterministic system, including period doublings (B) and transient oscillations in unstable limit cycles (C), which are however quite rare. (D) For very large amplitudes in the chaotic regime, trajectories starting from very similar initial conditions diverge quickly in time. The different colors show trajectories for initial conditions differing only in one molecule; they remain close for a while but eventually diverge exponentially. (E) Additional visualization of the data shown in (D) where the period between successive NF-κB peaks is plotted as a function of time. The horizontal lines correspond to integer multiples of the time period of the driving TNF oscillation. (F) Trajectory of oscillations in (D) in phase space for IKB, IKB_RNA_, and NK-kB. (G) Distribution of time periods for a simulation of 1,000 oscillations. The red indicates the distribution of periods for the deterministic simulation, and the blue indicates the distribution for stochastic simulation. Same parameters were used in the simulations. (H and I) Additional visualization of the structure in chaotic mode-hopping. The period to period correlation plot is shown in (H) and a transition heatmap (I) showing the probability of going from each entrained state to other entrained states, exhibiting no clear correlation between the jumps of states. (J) The number of transitions (over an interval of thousand oscillations) between entrained states for different noise levels, as a function of the external amplitude. Blue, V = 1 × 10 ^−15^ L; red, V = 2 × 10 ^−15^ L; green, V = 5 × 10 ^−15^ L; cyan, V = 15 × 10 ^−15^ L.

When the amplitude of the driving TNF oscillation is increased, we move up in the Arnold tongue diagram (Figure 4A), which leads the deterministic system into a chaotic regime (Jensen et al., 1984). Deterministic chaos is characterized by a trajectory in phase space that never repeats itself and has the property that two trajectories starting from slightly different initial conditions diverge exponentially in time (Lorenz, 1963). Chaotic states are reached for larger TNF amplitudes where many tongues overlap (Figure 4A). We characterized the behavior of the NF-κB oscillator near this region of parameter space.

As we increase the amplitude of the TNF oscillations, but before chaos sets in, a variety of interesting phenomena occur. For example, one of these known as period doubling, where it takes two oscillations peak NF-κB amplitude (Figure 4B). Even in the early onset of chaos, transient and unstable limit-cycle behaviors can be found (Figure 4C), but these are quite rare and disappear as we increase the amplitude of the TNF oscillations even further. Using the same tools we used to characterize noise-induced mode-hopping, if we study NF-κB oscillations in the chaotic system, that we observe oscillations starting in almost the same initial conditions will diverge after a few oscillations (Figure 4D). This is typical for chaotic systems and defined by the positive Lyapunov constant of the system. Reproducible tendencies, however, remain. When we study the periods of the NF-κB oscillator in period space under these conditions, we observe that even though they do not produce a clean pattern, they are always close to the integer values of the external periods, which are indicated by the lines (Figure 4E). This can be seen more clearly in the three-dimensional space spanned by NF-κB, I-κB mRNA, and I-κB, where we can see the trajectories are ordered in small bands (Figure 4F). Moreover, looking at 1,000 oscillations, we find that the distribution of periods is sharply peaked around integer multiples of the TNF period (Figure 4G). However, these behaviors are not reminiscent of mode-hopping as described above.

Next, we asked whether adding noise to the chaotic system could induce mode-hopping. We find that when the driving TNF oscillation is such that the deterministic system would exhibit chaos, then adding noise to our simulations does not reduce the entrainment of the NF-κB oscillations (Figure 4G). Moreover, for the high amplitude driving shown in Figures 4D–4F, we find that noise does produce trajectory hops between many entrained modes. When we plot the period-to-period correlation of these oscillators (Figure 4H), we find that all periods belong to well-defined tongues, as indicated by the layered structure of the plot. One might expect that the mode-hopping will occur between neighboring tongues, however, in Figure 4I, we show that jumps between distant tongues also occur frequently. In this sense, chaotic dynamics might be regarded as random transitions between various tongues, rather between specific oscillations with particular amplitudes and frequencies. Chaos and noise, therefore, both manifest as increasingly frequent mode-hopping as noise is increased or one moves deeper into the chaotic regime by increasing the amplitude of external TNF oscillations (Figure 4J). In fact, in the presence of noise, it is difficult to distinguish between the system being inside or outside the chaotic regime from the probability of exhibiting entrainment or the probability distribution of being in the various possible entrained states (Figure 4G). Notably, however, in the presence of noise, mode-hopping is already observed for small TNF amplitudes (Figure 3E) and is found for all higher TNF amplitudes, which is a much larger region of parameter space than the deterministic system, where chaos only sets in for larger amplitudes (Figure 4A).

There are important differences, however, between the dynamics of noise-induced mode-hopping below the transition into chaos and deterministic chaos above the transition. Comparing Figure 3E (noise-induced mode-hopping) and Figure 4E (mode-hopping within the chaotic regime), it is seen that the noise-induced mode-hopping only makes jumps between two states and usually remains in the same state for a few periods (Figure 3E), whereas the chaotic dynamics jumps between many different states and usually does not spend more than one period in each state (Figures 4E and 4J). These observations raise the question of whether the NF-κB mode-hopping seen in living cells is induced by noise or a function of a deterministic system operating above the transition to chaos. In experimentally observed NF-κB trajectories in living cells, we see that the system spends several periods in each entrained state and rarely visits more than two entrainment modes (Figure 2F and simulations from Figures 3E, 3H, and 3I). This suggests that, in experiments, the system sits in a region of parameter space where the Arnold tongues overlap but below the transition to chaos. More sophisticated ways exist to distinguish between chaos and randomness in dynamical trajectories (Amon and Lefranc, 2004), but we believe our arguments above are sufficient to suggest that the experimental NF-κB system has a relatively high level of noise and operates in the overlapping tongue region but below the transition to chaos.

### Mode-Hopping Enables Cells to Switch between High and Low Gene Production States

One potential advantage of oscillatory transcription factor dynamics is differential regulation of frequency-sensitive promoters. Frequency modulation and frequency-sensitive gene regulation occurs in the Crz1 system, ERK signaling, hormone regulation, and is speculated to exist in NF-κB immune signaling (Albeck et al., 2013; Ashall et al., 2009; Cai et al., 2008; Krishna et al., 2006; Mengel et al., 2010; Waite et al., 2009; Wee et al., 2012). Previously, Cai et al. (2008) showed that frequency modulation can ensure a proportional expression of multiple genes having different promoter characteristics. Our observations prompt the question: how could mode-hopping facilitate regulation of diverse frequency-sensitive genes?

**Figure 5:**
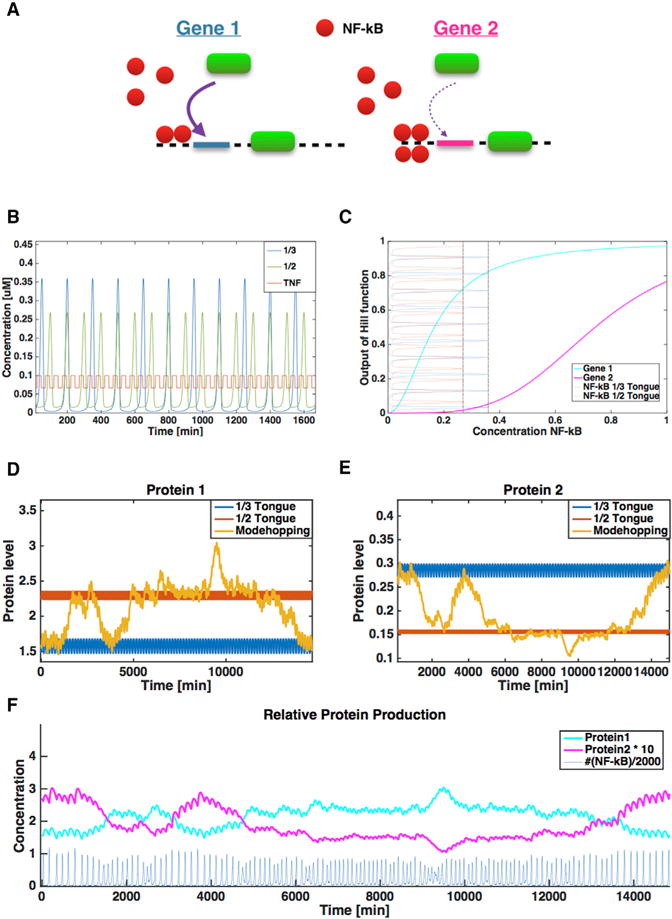
Mode-Hopping Switches between High and Low Gene Production States (A) Schematic figure of the downstream network for the two genes with distinct properties. The green oval represents RNA polymerase, which is recruited by NF-κB binding to a *cis*-regulatory region upstream of each gene. For gene 1, NF-κB binds to this region with high affinity and low cooperativity, while for gene 2 it binds with low affinity and high cooperativity. (B) NF-κB oscillation at two frequencies reflecting two different locking modes, tongue 1/2 and tongue 1/3. (C) Output of the Hill function for the mRNA production for each gene for a fixed level of NF-κB plotted as a function of NF-κB level. Oscillations from (B) are plotted vertically to indicate the range of NF-κB concentration oscillations in each tongue produce. (D and E) Plots of gene expression output for gene 1 (D) and gene 2 (E). (F) The expressed protein levels for the two different gene families. The cyan curve shows protein production for gene 1 and the magenta curve shows protein production for gene 2 (although multiplied by a factor 10). The blue shows the corresponding NF-κB oscillations used in to produce the protein production.

When oscillations of NF-κB switch between two tongues, frequency and amplitude of the oscillations change (Figure 5B), and this can alter the expression of different downstream genes that have NF-κB as a transcriptional regulator. Frequency-dependent NF-κB transcriptional regulation, in turn, may be achieved through altered binding affinity and cooperativity (Wee et al., 2012). As an example of this mechanism, we consider two genes, gene 1 and gene 2, regulated differentially by NF-κB (Figure 5A). NF-κB binds with high affinity and low cooperativity to the *cis*-regulatory region controlling expression of gene 1 and with low affinity and high cooperativity to the region controlling gene 2. The expression level of the two genes for different constant levels of NF-κB are shown in Figure 5C, along with the NF-κB oscillations in the 1/2 and 1/3 tongues (shown vertically) that demonstrates the differing range of NF-κB concentration produced during these oscillations (higher frequency results in a smaller maximum NF-κB level). Gene 1, having a higher affinity for NF-κB, has high expression for oscillations of both the frequencies shown in Figure 5C. In contrast, for the low affinity gene 2, Figure 5C shows that the expression level is low for the 1/2 tongue, because of its lower amplitude oscillations, and substantially higher for the 1/3 tongue that has a higher amplitude. In Figures 5D and 5E, the protein production from gene 1 and gene 2 is plotted as a function of time for each individual tongue and in the case of mode-hopping. Figure 5F shows that, in contrast to constant regulation across multiple genes, mode-hopping allows different regulation across different frequency-sensitive promoters at different times. A list of the applied parameter values can be found in the second table of the STAR Methods.

The cell’s ability to switch between high and low production states for different, defined subsets genes, as shown in Figure 5F, is what we define here as “multiplexing.” The mechanism could, in principle, act together with, or in addition to, other mechanisms of multiplexing. Such mechanisms may allow the cell to dedicate its resources to producing one specific gene/protein at a given time, rather than a broad repertoire of genes/proteins at a time. Even though of random nature, this mode-hopping can be controlled in a statistical way by the cell. Changing the frequency or amplitude of TNF will change the position in the Arnold tongues and thus the probability of being in one state as opposed to the other. For instance, a TNF with amplitude below overlap of Arnold tongues would stay in one state, while going to an overlap with competition between different states, would allow for frequent mode-hopping. In this way, the cell can use the Arnold tongues to upregulate the time in different states without completely losing the possibility of jumping between states. We note that this mechanism is not necessarily the only, or even the main, functional effect of noise in protein dynamics inside the cell but rather points out how this stochastic nature can be used in an advantageous and regulatory way.

## DISCUSSION

Oscillations in gene regulatory networks are known to control transcriptional specificity and efficiency (Kellogg and Tay, 2015; Levine et al., 2013; Wee et al., 2012). We have shown here experimentally that entrained NF-κB oscillations in single cells exhibit jumps in frequency under high amplitude fluctuating TNF stimulation, a phenomenon we called “mode-hopping.” During these frequency jumps, cells maintain entrainment with the TNF input; this suggests that the system functions in the region of overlapping Arnold tongues. Previous studies have demonstrated that well entrained oscillations result in certain genes having higher expression (Kellogg and Tay, 2015). Within the overlapping Arnold tongue region of parameter space, a gene may exhibit two types of entrained oscillations, which we call entrainment modes. The presence of multiple entrainment modes may diversify biological functions. For example, oscillatory transcriptional control is using frequency modulation to control gene expression output and specificity (Ashall et al., 2009; Cai et al., 2008). Genes differ in affinity and cooperativity characteristics, which consequently determines sensitivity to frequency and amplitude of NF-κB regulation (Figure 5A). Therefore, changing NF-κB entrainment states causes switching between high and low gene production over time. For genes that are differentially sensitive to NF-κB frequency and amplitude, mode-hopping switches activation on and off for multiple genes over time (Figure 5E). This temporally multiplexed gene regulation contrasts to regulation under unchanging NF-κB oscillation, which drives expression across multiple genes equally over time.

This work uncovers a function for noise in gene regulation that, to the best of our knowledge, has not been previously reported. NF-κB activates hundreds of genes, requiring mechanisms for controlling relative expression level and specificity under fluctuating environmental signals. As we show, noise-induced jumps in NF-κB oscillation frequency can cause temporal switching between genes with diverse promoter characteristics over time. This method of gene regulation could facilitate management of amino acid or other metabolic factors by dedicating resources to synthesis of a defined subset of proteins at one time. Cellular mode-hopping therefore expands the toolbox of single cells to control the dynamics, specificity, and efficiency of gene expression and protein production.

## STAR★METHODS

Detailed methods are provided in the online version of this paper and include the following:

### KEY RESOURCES TABLE

**Table:** 

REAGENT or RESOURCE	SOURCE	IDENTIFIER
Chemicals, Peptides, and Recombinant Proteins		
TNF	Life Technologies	PMC3014
Experimental Models: Cell Lines		
p65-DsRed/H2B-GFP 3T3 mouse fibroblasts	Tay et al., 2010	N/A
Software and Algorithms		
ROOT	Brun and RAdemakers, 1997	https://root.cern.ch/documentation
Simulations made in c++	This Paper	
MATLAB 6.1	The MathWorks Inc. 2010	https://se.mathworks.com/products/matlab/
Gillespie Algorithm	Gillespie, 1977	
Cellprofiler	http://cellprofiler.org/	
Other		
Automated microfluidic cell culture system	Kellogg et al., 2014	N/A
DMEM	Life Technologies	cat. no. 32430-027
FBS	Sigma-Aldrich	cat. no. F2442-500ML

### CONTACT FOR REAGENT AND RESOURCE SHARING

Further information and requests for reagents may be directed to, and will be fulfilled by the corresponding author Mogens Høgh Jensen (mhjensen@nbi.ku.dk).

### EXPERIMENTAL MODEL AND SUBJECT DETAILS

Mouse (3T3) fibroblasts expressing near-endogenous p65 levels were described previously (Tay et al., 2010; Kellogg and Tay, 2015). Briefly, p65−/− mouse 3T3 fibroblasts were engineered to express p65-DsRed under control of 1.5kb p65 promoter sequence (Tay et al., 2010). The cell line was clonally derived to express at p65-DsRed at lowest detectable level to preserve near endogenous expression (Tay et al., 2010). Addition of ubiquitin-promoter driven H2B-GFP expression provided a nuclear label to facilitate automated tracking and image processing.

### METHOD DETAILS

#### Cell Culture and Live Cell Imaging

Automated microfluidic cell culture and periodic TNF stimulation was performed as previously described (Kellogg et al., 2014; Tay et al., 2010; Kellogg and Tay, 2015). In vitro cultures were maintained in DMEM (Life Technologies, cat. no. 32430-027) and FBS (Sigma-Aldrich, cat. no. F2442-500ML). Prior to seeding in the microfluidic device, NIH 3T3 fibroblasts were cultured in (DMEM + 10% (vol/vol) FBS). Cells were passaged 1:10 every 3 days to not exceed 80% confluency. Standard culture conditions of 5% CO2 and 37°C were maintained using an incubation chamber during culturing and throughout imaging experiments.

Briefly the live cell microscopy experiments proceeded as follows: microfluidic chambers were fibronectin treated and seeded with cells at approximately 200 cells/chamber. Cells were allowed to grow for one day with periodic media replenishment until 80% confluence. To stimulate cells, media equilibrated to 5% CO2 and containing the desired TNF amount was delivered to chambers, leading to a step increase in TNF concentration. To produce periodic TNF signals, chambers were washed with media containing TNF at the desired intervals. Chambers were imaged at 5–6 min intervals. DsRed and GFP channels were acquired using a Leica DMI6000B widefield microscope at 20x magnification with a Retiga-SRV CCD camera (QImaging) using Leica L5 and Y3 filters to acquire GFP and DsRED signals, respectively and a Leica EL6000 mercury metal halide light source.

#### Mathematical Modeling

We consider the model, previously published by Jensen and Krishna (2012), of the NF-κB, defined by the 5 coupled differential equations given as:

$$\frac{dN_{n}}{dt}=k_{\mathrm{Nin}}(N_{\mathrm{tot}}-N_{n})\frac{K_{I}}{K_{I}+I}-k_{\mathrm{lin}}I\frac{N_{n}}{K_{N}+N_{n}}$$

$$\frac{dI_{m}}{dt}=k_{t}N_{n}^{2}-\gamma_{m}I_{m}$$

$$\frac{dI}{dt}=k_{tI}I_{m}-\alpha {\left[\mathrm{IKK}\right]}_{a}(N_{\mathrm{tot}}-N_{n})\frac{I}{K_{I}+I}\;.$$

$$\frac{d{\left[\mathrm{IKK}\right]}_{a}}{dt}=k_{a}[\mathrm{TNF}]({\left[\mathrm{IKK}\right]}_{\mathrm{tot}}-{\left[\mathrm{IKK}\right]}_{a}-{\left[\mathrm{IKK}\right]}_{i})-k_{i}{\left[\mathrm{IKK}\right]}_{a}$$

$$\frac{d{\left[\mathrm{IKK}\right]}_{i}}{dt}=k_{i}{\left[\mathrm{IKK}\right]}_{a}-k_{p}{\left[\mathrm{IKK}\right]}_{i}\frac{k_{A20}}{k_{A20}+[A20][\mathrm{TNF}]}$$

The background and the underlying assumptions for this model, is previously published and the relevant discussions in this regard are presented in that paper (Jensen and Krishna, 2012). All the parameters in the model is seen in the table below. The first nine are from Krishna et al. (2006) and the following four from Ashall et al. (2009).



ParameterDefault value
k_Nin_5.4 min^−1^
k_Iin_0.018 min^−1^
k_t_1.03 (μM) · min^−1^
k_tl_0.24 min^−1^
K_I_0.035 μM
K_N_0.029 μM
γ_m_0.018 min^−1^
α1.05 (μM) · min^−1^
N_tot_1.0 μM
k_a_0.24 min^−1^
k_i_0.18 min^−1^
k_p_0.036 min^−1^
k_A20_0.0018 μM
[IKK]_tot_2.0 μM
[A20]0.0026 μM


#### Multiplexing Model

Protein and mRNA production by these genes is described by the following equations:

$${\dot{m}}_{i}=\gamma_{i}\frac{N^{h_{i}}}{K_{i}^{h}+N^{h_{i}}}-\delta_{i}m_{i}.$$

$${\dot{P}}_{i}=\Gamma_{i}m_{i}-\Delta_{i}P_{i}$$

Here the *m_i_* represents the mRNA of species *i*, and *P_i_* represents the protein level of species *i*. As can be seen from Figure 5A, the two genes differ only in two parameters, the affinity of the binding represented by K*_i_* and the cooperativity represented by hill coefficient *h_i_*. γ_i_ describes the mRNA production per time, δ_i_ is the decay of mRNA per time, Γ_i_ is the protein production per time and Δ_i_ is the decay of the protein per time. All parameters in this model is found in the table below:



Default Value
Default Value
ParameterGene 1Gene 2
K1.0 #molecules1.0 #molecules
h2.04.0
γ4.0 #molecules · min^−1^4.0 #molecules · min^−1^
Γ2.0 min^−1^2.0 min^−1^
δ2.0 min^−1^2.0 min^−1^
Δ0.3 min^−1^0.3 min^−1^

### QUANTIFICATION AND STATISTICAL ANALYSIS

CellProfiler software (http://cellprofiler.org) and custom MATLAB software was used to automatically track cells and quantify NF-κB translocation, and automated results were manually compared with images to ensure accuracy prior to further analysis. NF-κB activation was quantified as mean nuclear fluorescence intensity normalized by mean cytoplasm intensity. For peak analysis data were smoothed (MATLAB function *smooth*) followed by peak detection (MATLAB function *mspeaks*). Peaks were filtered based on reaching a threshold 10% of maximum intensity.

### DATA AND SOFTWARE AVAILABILITY

#### Software

All simulations were performed using scripts written in c++ and MATLAB. All data-analysis were performed from scripts written in python and using the ROOT software.

All scripts used for simulation and data analysis from the model, will be available upon request to Mathias Luidor Heltberg (heltberg@nbi.ku.dk)

#### Algorithms

All deterministic simulations were performed using Runge-Kutta 4th order simulations. All stochastic simulations were performed using the Gillespie algorithm (Gillespie, 1977). We considered 10 possible reactions given from the 10 different terms in the 5 differential equations.

## In Brief

Oscillations and noise drive many processes in biology, but how both affect the activity of the transcription factor NF-κB is not understood. This paper describes “cellular mode-hopping,” phenomenon in which NF-κB exhibits noise-driven jumps between defined frequency modes. The authors suggest that mode-hopping is a mechanism by which different NF-κB-dependent genes under frequency control can be expressed at different times.

## Highlights

- Periodic TNF stimulation leads to hopping between NF-κB entrainment modes
- For regions of overlapping Arnold tongues, intrinsic noise leads to mode-hopping
- Chaotic dynamics is characterized by frequent mode-hopping
- Mode-hopping suggests mechanism for the cell to regulate protein production
